# Clinical Management of Epilepsy With Glutamic Acid Decarboxylase Antibody Positivity: The Interplay Between Immunotherapy and Anti-epileptic Drugs

**DOI:** 10.3389/fneur.2018.00579

**Published:** 2018-07-13

**Authors:** Kari-Matti Mäkelä, Aki Hietaharju, Antti Brander, Jukka Peltola

**Affiliations:** ^1^Department of Neurology, University of Tampere, Tampere University Hospital Tampere, Finland; ^2^Department of Radiology, Medical Imaging Centre, Tampere University Hospital Tampere, Finland

**Keywords:** clinical management, glutamic acid decarboxylase antibody, limbic encephalitis, autoimmune epilepsy, case series

## Abstract

**Background:** There is scanty guidance in the literature on the management of patients with glutamic acid decarboxylase (GAD65) antibody associated autoimmune epilepsy (GAD-epilepsy). GAD-epilepsy is a rare distinct neurological syndrome with a wide clinical spectrum. We describe six GAD-epilepsy patients with special emphasis on the treatment timing and the relationship between immunologic and anti-epileptic therapy.

**Methods:** Six patients diagnosed with GAD-epilepsy in Tampere University Hospital who had received immunotherapy from 2013 to 2017 were retrospectively analyzed from patient records. Data about symptom onset, including antibody levels, magnetic resonance imaging (MRI), electroencephalograms, immunotherapy and anti-epileptic treatment timing and treatment responses were collected and analyzed. Kruskall-Wallis test was used in the statistical evaluation.

**Results:** All patients were female aged 9–54 at symptom onset. Three had hypothyroidism, none had diabetes, two had migraine. Five patients had very high (>2,000 IU/ml) and one had high (52–251 IU/ml) GAD65 antibody titers. All patients presented with seizure disorders. Patients who received early initiation of immunotherapy (3–10 months) responded well to treatment; patients in whom the immunotherapy was started later (15–87 months) did not respond (*p* = 0.0495). The first patient was seizure-free after 1 year of regular intravenous immunoglobulin and one antiepileptic drug (AED). The second patient developed unilateral temporal lobe T2 signal changes in MRI; she responded well to immunotherapy, experiencing a significant reduction in seizure frequency and resolution of MRI abnormalities. However, seizures continued despite trials with several AEDs. The third patient responded well to immunoadsorption and rituximab with one AED, with lowering of GAD65 titers (from >2,000 to 300). There was a long delay in the diagnosis of GAD-epilepsy in the three patients who had developed refractory epilepsy, one with hippocampal sclerosis. They all received immunotherapy but none responded. However, AED modification or vagus nerve stimulation reduced the seizure frequency in two patients. Epilepsy surgery was ineffective.

**Conclusions:** These results highlight the importance of early detection of GAD65 antibodies in refractory epilepsy as immunotherapy can be effective if administered in the early stages of the disease when it can prevent permanent brain tissue damage.

## Introduction

Autoimmunity is increasingly being recognized as a cause of epilepsy (1). Glutamic acid decarboxylase 65-kilodalton isoform (GAD65) antibodies have been associated with multiple non-neurological and neurological syndromes including autoimmune epilepsy (2).

GAD65 is an intracellular antigen, highly expressed in the presynaptic terminals of inhibitory neurons in the central nervous system (CNS) and in pancreatic β-cells (3). GAD65 antibodies possibly serve as a surrogate marker for organ specific autoimmune disorders mediated by cytotoxic T cells (4). However, there might also be some currently unknown pathogenic surface-antigens targeted against hippocampi co-existing with the GAD65 antibody and contributing to temporal-lobe epilepsy (TLE) (5). Furthermore, the related pathological processes can lead to hippocampal sclerosis and refractory epilepsy (6). Moreover, widespread white matter changes have been observed in GAD65 antibody related limbic encephalitis (LE) (7).

Recently, anti-neuronal antibodies were detected in 20.5% of epilepsies of unknown etiology and of these, 64% were high titer GAD65 antibodies (8). Previously, it has been estimated that between 1.7% (9) and 8.7% (10) of epilepsy patients are harboring GAD65 antibodies.

GAD65 antibody associated autoimmune epilepsy (GAD-epilepsy) is a rare but distinct neurological syndrome with a wide clinical spectrum ranging from mild non-pharmacoresistant epilepsy (10) to refractory TLE (11), LE (12), and also extra-limbic encephalitis (ELE) (13). It seems that indolent GAD65 autoimmunity can develop into more severe forms over time (14).

The literature contains only a few case reports dealing with the management of refractory GAD-epilepsy (15). In addition to anti-epileptic drugs (AEDs), a plethora of immunotherapies has been tried with variable or unsatisfactory results (11, 15, 16). Overall, the response to immunotherapy is poor and only a few patients achieve seizure-freedom (17).

Since there is no clear guidance in the literature with respect to the timing or on the combination of immunotherapy with AEDs in the management of GAD-epilepsy, here we describe six GAD-epilepsy cases treated with immunotherapy during different disease stages and compare the results of immunotherapy with those achieved by AEDs.

## Materials and methods

### Study cohort

Patients treated in Tampere University Hospital Department of Neurology for GAD-epilepsy between the years 2012 and 2017 were studied. The clinical data was analyzed retrospectively from patient records. The initial diagnosis was suspected due to the clinical symptoms and then supported by highly elevated titers of serum GAD65 antibodies. Written informed consent was obtained from the participants for the publication of this case series.

### Statistics

All statistical calculations were done in R version 3.4.3 (www.r-project.org). Kruskall-Wallis test was used to compare treatment results in immunotherapy responders vs. non-responders.

### Laboratory and imaging studies

GAD65 antibody levels were analyzed in Fimlab laboratories (Tampere, Finland) with standard clinical methods. In most patients, Euroimmun (Luebeck, Germany) anti-GAD ELISA (IgG) was used according to the manufacturer's protocol. Prior to 2014, the Medizym (Berlin, Germany) anti-GAD ELISA (IgG) was used according to the manufacturer's protocol. Most serum and cerebrospinal fluid (CSF) neuronal autoantibody panels were determined in Wieslab (Malmö, Sweden) with standard methods. In patient 1, CSF neuronal antibodies were analyzed in the Institut D'Investigacions Biomédiques August Pi I Sunyer, (Hospital Clinic, University of Barcelona, Spain). Other laboratory studies were undertaken with standard laboratory methods at Fimlab laboratories. Brain magnetic resonance images (MRI) were obtained according to a dedicated epilepsy protocol on a 3 Tesla scanner. Electroencephalograms (EEG) were obtained with standard protocols.

### Therapeutic interventions

Immunotherapy, including immunoadsorption, was administered in all patients by following generally accepted clinical principles. Accordingly, AED treatment was provided to all patients in order to achieve maximum seizure control and tolerability. Selective amygdalo-hippocampectomy (SAH) and vagus nerve stimulator (VNS) were offered to some drug-resistant patients after they had undergone comprehensive pre-surgical diagnostics according to the current standards.

### Treatment outcomes

Outcome variables were the seizure or other main symptom frequencies estimated from patient records such that an over 50% symptom reduction was considered as a good treatment response; changes in the GAD65 antibody titer levels were also determined.

## Results

All six patients were female aged 9–54 at symptom onset (Table 1) and presented with seizure disorders (Table 2). Patients 1–3 displayed a positive response whereas patients 4–6 exhibited a negative response to immunotherapy; in the former group, the mean delay from symptom onset to immunotherapy initiation was only 5.7 months (range = 3–10 months) whereas in the latter group, it was significantly longer, 66 months (range = 15–87 months) *p* = 0.0495.

**Table 1:** Individual patient characteristics, serological and cerebrospinal fluid studies.

	**Patient 1**	**Patient 2**	**Patient 3**	**Patient 4**	**Patient 5**	**Patient 6**
Age at onset, years	54	19	20	9	14	16
Sex	Female	Female	Female	Female	Female	Female
Symptom onset	2014/2	2012/7	2014/6	2007/12	2011/3	2014/1
Immunotherapy initiated	2014/5	2013/5	2014/10	2015/3	2012/6	2016/3
Comorbidities	Hypothyroidism	Migraine	Hypothyroidism, migraine	Hypothyroidism	-	-
GAD65 ab, serum, IU/ml	52–251	over 2,000	over 2,000	over 2,000	over 2,000	over 2,000
GAD65 ab, CSF	Negative	Positive	Negative	Negative	Positive	Not done
Serum studies, positive	TPO, VGKC (low), B2GP (low)^*#£$%″^	All negative^¤$#+*x*^	All negative^μ#−*x*&*z*^	ICA (5120 IU/ml)^*£#∧*i*^	ANA^*#£∧*x*&^	ANA^*#″*c*^
CSF studies, positive	VGKC (low)^*^	All negative^¤!^	All negative^μ^	All negative^*^	All negative^*^	All negative^*^
CSF (WBC, protein, IgG-index, oligoclonal bands)	1, 1443-923, elevated, no	11-3, normal, elevated, yes	All normal	Normal, normal, normal, yes	Normal, elevated, elevated, yes	6, normal, normal, yes

*The individual laboratory studies are indicated with superscripts; only positive results are shown*.

ab, antibody; aCL, anticardiolipin ab; ANA, anti-nuclear antibody; ANCA, anti-neutrophil cytoplasmic antibody; AMPA, α-amino-3-hydroxy-5-methyl-4-isoxazolepropionic acid; B2GP, Beta-2 Glycoprotein 1 Antibodies; caspr2, contactin-associated protein-like 2; CCP, cyclic citrullinated peptide ab; CSF, cerebrospinal fluid; C, complement; DNA, Deoxyribonucleic acid; ENA, Extractable nuclear antigen; GABA, gamma-aminobutyric acid; GAD65, Glutamic acid decarboxylase 65-kilodalton isoform; HbA1c, Hemoglobin A1c; HHV, Human herpesvirus; HIV, Human immunodeficiency virus; HSV-PCR, herpes simplex virus polymerase chain reaction; ICA, islet cell antibodies; LGI1, leucine-rich glioma inactivated 1; mGluR, metabotropic glutamate receptor; NMDA, N-Methyl-D-aspartate receptor; MPO, myeloperoxidase ab; PR3, anti-proteinase 3; RF, rheumatoid factor; RNP, ribonucleoprotein; SSA, anti-Sjögren's-syndrome-related antigen A; SSB, anti-Sjögren's-syndrome-related antigen B; TPO, thyroid peroxidase; TSH, thyreotropin; TTGA, Tissue transglutaminase ab; VGKC, voltage gated potassium channel; WBC, white blood cells;

* AMPA-1, caspr2, GABA-B, LGI1, mGluR1, mGluR5, NMDA;

# ampiphysin, ANA, ANCA, DNA, ENA

$ borrelia, aCL, B2GP;

£ CV2, Hu, Ma1, Ma2, Ri, Sox1, Yo;

¤ NMDA,VGKC, AMPA-1, GABA-B, HHV-6;

μ NMDA, VGCK;

% HIV, 14-3-3;

+ TTGA;

− MPO;

″ TPO;

∧ RNP, SSA, SSB;

i ICA

! HSV-PCR;

x C3, C4;

& RF;

z CCP, HbA1c, TSH, thyroxine;

c *cryoglobulin*.

**Table 2:** Seizure types, imaging studies, treatments, and treatment responses.

	**Patient 1**	**Patient 2**	**Patient 3**	**Patient 4**	**Patient 5**	**Patient 6**
Seizure types (18)	NCSE	FIAS	FBTCS, FIAS, FAS	FIAS	FIAS	FBTCS, FAS
Other symptoms	Fast cognitive decline, vertigo, tremor, dystonia, aphasia, hallucinations.	Memory defect, depression, vertigo.	Headache, cognitive impairment, tremor, anxiety, left sided weakness.	Cognitive slowing, nausea, depression.	Memory problems, compulsive thoughts, fear, anxiety.	Eczema, joint symptoms.
Epilepsy type	Focal (onset unknown)	Focal (bitemporal)	Focal (temporal)	Focal (bitemporal)	Focal (bitemporal)	Focal (multifocal)
EEG	During the SE episode slow wave discharges bilaterally with frontal maximum.	Ictally left or right temporal lobe discharges.	Interictal normal, no ictal recordings.	Ictally left or right temporal lobe discharges.	Ictally left or right temporal lobe discharges.	Ictally left or right widespread discharges without definitive localizing features.
MRI	Normal	Left temporomesial T2 signal change which resolved after treatment.	Normal	Normal	Left hippocampal sclerosis.	Marginal right hippocampal atrophy.
Immunotherapies	IVIg, MP, PR, RTX, MMF	IVIg, MP, PR, MMF, RTX, IA	IVIg, MP, IA, RTX	IVIg, MP, IA, RTX	IVIg, AZP, IA, RTX	IVIg, HCQ
Current AEDs	LCM	CBZ, LCM, LEV, ZNS	TPM	OCZ, ZNS	AZM, LCM, ZNS	ECZ, LEV, OCZ
Prior AEDs	CBZ, LEV, LZP, PEH, TPM, VLP, CLB	OCZ	–	LEV	CBZ	CBZ, CLB, LEV, OCZ, PRG, VLP
Epilepsy surgery	No	No	No	Yes, left temporal lobe, no HS. VNS.	Yes, left temporal lobe, HS.	No
Treatment response	Symptom-free after 1 year with regular IVIG.	Good response to IA, nevertheless refractory epilepsy.	Good response to IA and RTX, however multiple relapses.	No response to late immunotherapy, response to VNS.	No response to late immunotherapy, surgery or AED.	No response to late immunotherapy, response to AED.

*AED, anti-epileptic drug; AZM, acetazolamide; AZP, azathioprine; CBZ, carbamazepine; CLB, clobazam; CP, Cyclophosphamide; ECZ, eslicarbazepine; EEG, Electroencephalography; FAS, Focal aware seizure; FBTCS, Focal to bilateral tonic–clonic seizure; FIAS, Focal impaired awareness seizure; HS, hippocampal sclerosis; HCQ, hydroxychloroquine; IA, immunoadsorption; IVIg, intravenous immunoglobulin; LCM, lacosamide; LEV, levetiracetam; LZP; lorazepam; MMF, mycophenolate mofetil; MP, methylprednisolone; MRI, Magnetic resonance imaging; NCSE, non-convulsive status epilepticus; OCZ, oxcarbazepine; PEH, phenytoin; PR, prednisolone; PRG, pregabalin; RTX, rituximab; TPM, topiramate; VNS, vagus nerve stimulation; VLP, sodium valproate; ZNS, zonisamide*.

**Figure 1:**
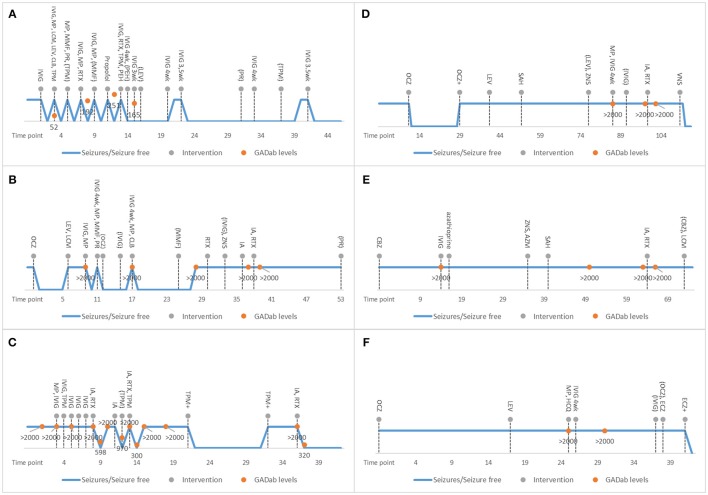
Individual characteristics of treatment responses and therapies provided in the studied GAD-epilepsy patients are shown. X-axis shows the time-points in months starting from symptom onset. The blue line displays seizures / no seizures. Dotted lines refer to the interventions. Orange dots are GAD65 antibody levels. Discontinuation of therapies is shown in parenthesis. 4 wk means 4-week intervals. Patient 1 **(A)** responded well to early initiation of immunotherapy. With patient 2 **(B)**, there was longer delay before immunotherapy and she continued to experience seizures even after several AED and immunotherapy trials. However, her MRI pathology resolved. Patient 3 **(C)** responded well to immunoadsorption with decreasing of GAD65 antibody levels after every trial. Patients 4–6 **(D–F)** did not respond to late immunotherapy. In patient 4, a vagus nerve stimulator ultimately reduced seizure levels. In patient 6, AED modification reduced her seizure levels. AED, antiepileptic drugs; AZM, acetazolamide; AZP, azathioprine; CBZ, carbamazepine; CLB, clobazam; CP, Cyclophosphamide; ECZ, eslicarbazepine; GAD65, Glutamic acid decarboxylase 65-kilodalton isoform; HCQ, hydroxychloroquine; IA, immunoadsorption; IVIG, intravenous immunoglobulin; LCM, lacosamide; LEV, levetiracetam; LZP; lorazepam; MMF, mycophenolate mofetil; MP, methylprednisolone; OCZ, oxcarbazepine; PEH, phenytoin; PR, prednisolone; RTX, rituximab; SAH, selective amygdalohippocampectomy; TPM, topiramate; VNS, vagus nerve stimulation: wk, week; VLP, sodium valproate; ZNS, zonisamide;.

A 54-year-old woman (patient 1; Figure 1A) presented in the emergency department with a few weeks' history of cognitive decline and fluctuating vertigo, aphasia and tremor. The neurological examination detected a fine tremor in all limbs and total aphasia. The EEG revealed non-convulsive status epilepticus (NCSE) without definitive lateralizing or localizing features and this was treated with intravenous immunoglobulin (IVIg) and IV AEDs. The NCSE resolved within 24 h. However, she experienced several relapses which mostly started with speech difficulties leading to total aphasia, confusion, anxiety, mild gait abnormality and tremor. NCSE relapsed three times and of these two were treated successfully with IVIg. One NCSE was successfully treated with propofol. Ultimately, the patient was suffering only a mild speech impairment and gait disturbance at the end of her immunotherapy cycle. Because of no relapses for 3 years with IVIg, the gradual reduction of dosage and increase of treatment interval is ongoing. The patient is still on AED monotherapy.

**Figure 2:**
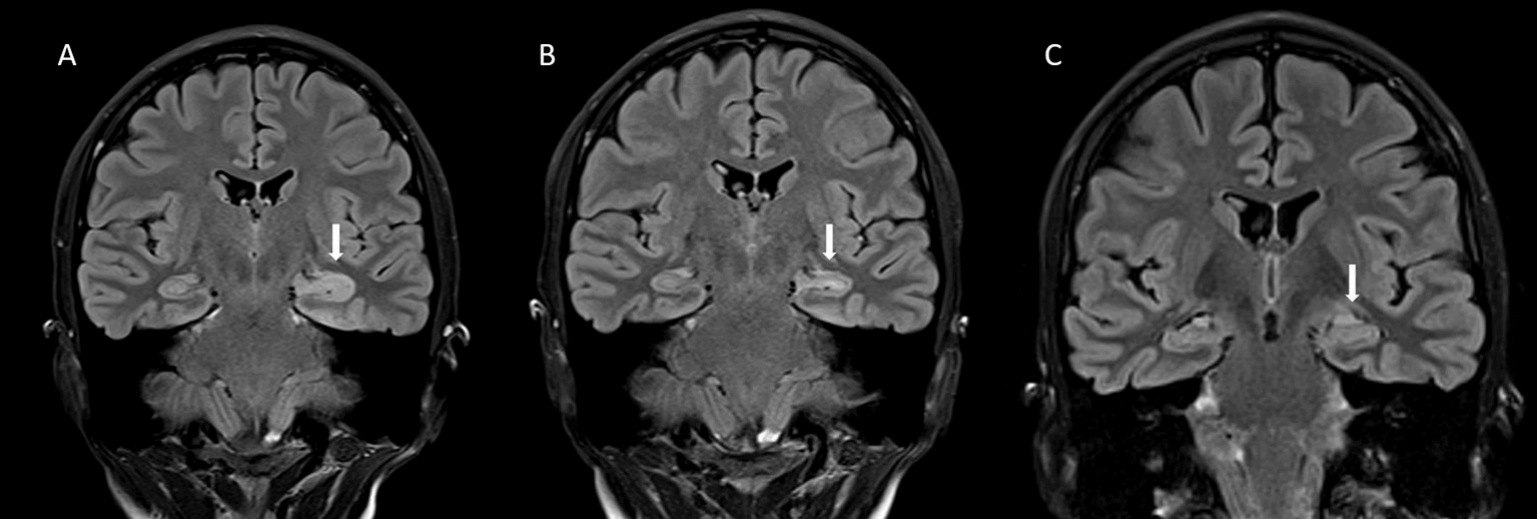
**(A)** The coronal fluid attenuation inversion recovery (FLAIR) magnetic resonance (MRI) -image taken during the acute stage of the illness shows an abnormally hyperintense and swollen head of the left hippocampus (arrow). **(B)** Five months later, the finding has mostly resolved, although slight hyperintensity of the left hippocampal head can still be seen (arrow). **(C)** In a control image, 3 years and 8 months after the acute stage, the abnormal finding has totally resolved (arrow). There are no signs of atrophy in the primarily affected area.

A 19-year-old woman (patient 2; Figure 1B) was brought to the emergency department with daily focal impaired awareness seizures (FIAS) (18, 19) and complaints of memory impairment. TLE was diagnosed and the patient was almost symptom-free for 6 months with one AED, experiencing only mild aura symptoms once a month. Her seizure frequency increased and a second AED was initiated but with no clear response. GAD-epilepsy was diagnosed during further examinations and her response to immunotherapy was dramatic, resulting in almost complete resolution of seizures. A follow-up MRI revealed a novel left temporomesial signal change and edema correlating with the EEG findings (Figure 2). In later follow-up MRIs after repeated immunotherapy, the signal changes had started to resolve and in due course, disappeared completely. A mild memory impairment was confirmed in the neuropsychological examination; this did not respond to immunotherapy. The patient continued to have only a few FIAS daily. Immunotherapy was eventually terminated since it did not provide any further reduction in her seizure activity and the MRI abnormalities had resolved. This caused neither increase in seizure frequency nor worsening of her condition. She is still experiencing regular FIAS and is being treated with four AEDs.

A 20-year-old woman (patient 3; Figure 1C) presented in the emergency department after focal to bilateral tonic-clonic seizures (FBTCS). On arrival, she had mild left sided weakness and aphasia which soon resolved and she was discharged. For a few weeks before the seizure, she had experienced mild cognitive symptoms, mainly confusion. Headache, left-sided weakness and the feelings of confusion relapsed without there being any seizures. GAD-epilepsy was diagnosed early and immunotherapy initiated to prevent worsening of the symptoms. AED was provided mainly for migraine prevention. There was no clear response to the initial immunotherapies and they had to be stopped due to adverse effects. The patient started to suffer anxiety and fear-like emotions after a second FBTCS. She was provided with secondary immunotherapy with immunoadsorption (IA) and there was clear resolution of symptoms and also a lowering of GAD65 antibody levels. However, she continued to experience focal unaware seizures (FAS) with mild right sided arm twitching and there was a return of the high GAD65 antibody titer levels; therefore, IA was repeated with a good response.

A 9-year-old girl (patient 4; Figure 1D) presented with nausea, abdominal pain and excessive swallowing and TLE was diagnosed. She was symptom-free with one AED for 1 year until she started to have 40 FIAS on a monthly basis. Multiple AEDs and epilepsy surgery did not reduce her seizure frequency. High GAD65 antibody levels were detected when performing an extensive serology panel before VNS implantation 7 years after symptom onset. Since primary immunotherapy achieved no effects, secondary immunotherapy with IA and rituximab was tried but with no symptom relief and no effect on GAD65 antibody levels. Immunotherapy was discontinued and a VNS implanted, which when combined with two AEDs, achieved an initial response, i.e., the patient became seizure-free.

A 14-year-old girl (patient 5; Figure 1E) presented with FIAS and TLE was diagnosed. Brain MRI revealed left hippocampal sclerosis. Multiple AEDs and epilepsy surgery did not reduce her seizure frequencies. GAD-epilepsy was diagnosed 15 months after symptom onset. She received primary immunotherapy but it offered no benefits. Some years later, IA and rituximab were tried but these neither eased her symptoms nor reduced her antibody levels. She is still experiencing regular FIAS despite therapy with three AEDs.

A 16-year-old girl (patient 6; Figure 1F) presented with FBTCS, eczema and joint pain. Despite treatment with two AEDs, she continued to experience FIAS and high serum GAD65 antibody levels were detected 26 months after symptom onset. Primary immunotherapy had no effect on seizures and it was discontinued due to adverse effects. Hydroxychloroquine eased her joint symptoms and this therapy was continued but she still experienced FIAS. With AED modification, her seizure levels declined and thus secondary immunotherapy was not tried.

## Discussion

We have described the clinical management of six patients with GAD-epilepsy. Three patients responded well to early immunotherapy initiated within 10 months after symptom onset and one patient's brain MRI abnormalities resolved after regular immunotherapy. Immunotherapy achieved no objective benefit in three patients who already had developed refractory epilepsy. Instead, AED modification or VNS implantation achieved better clinical results than immunotherapy in patients in whom the diagnosis of GAD-epilepsy had been delayed. Epilepsy surgery was ineffective in these patients.

Even though the biological process is most likely a continuum, our results suggest that the clinical course of GAD-epilepsy forms three major stages. In the first stage, reversible acute immunoactivation causes the first seizure (20). In this stage, the main focus of management should be placed on immunotherapy since this can prevent permanent brain tissue damage and stop the epilepsy from becoming refractory, as was seen with patients 1 and 3. In the second stage of GAD-epilepsy, there is already subtle irreversible brain tissue damage (4), which causes refractory epilepsy (Patient 2). During the second stage, immunotherapy can still be highly effective as was seen with the resolution of brain MRI abnormalities in patient 2. However, it seems that after the resolution of the immunoactivation, the focus in management should shift to managing the refractory epilepsy. In the third stage, there has been progressive damage leading to hippocampal sclerosis and to a more diffuse brain damage and cognitive symptoms (7). In this stage, immunotherapy seems to be ineffective and the emphasis should be on the management of the refractory epilepsy.

All of the evidence surrounding the management of GAD-epilepsy has been based on small case reports and the treatment results have been variable (15). The patients in our study largely resemble previous study populations with a female sex predominance and young age. In patients with diabetes, GAD65 antibody titer levels of over 200 IU/ml are considered high (21). In GAD-epilepsy, both high and very high (over 1,000 IU/ml) GAD65 antibody titer levels have been detected (2) which is in accordance with the findings in our patients. CSF was abnormal in all but one of our patients. Especially patients 2 and 5 showed significant immunoactivation in the CSF. Malignancy is rarely associated with GAD-epilepsy (15) as was also shown in our data. Many GAD65 antibody positive patients harbor other autoantibodies indicative of polyautoimmunity (22). Accordingly, two of our patients had ANA and one harbored TPO-antibodies. GAD-epilepsy patients can also develop diabetes or other neurological GAD65 antibody associated syndromes (3) although this was not observed in our patients. Even in non-diabetic patients, the GAD65 antibody positivity is strongly associated with thyroid disease (23) which was also present in 50% of our patients. Patient 1 had low titer antibodies against the VGKC complex but tested negative for Caspr2 and LGI1. This finding is of uncertain clinical value (24). In our previous study, we did not detect the presence of VGKC antibodies in GAD-epilepsy patients (25).

In most case reports, IVIg and MP are the standard first line immunotherapies administered (11, 15, 26) in GAD-epilepsy as was the case with our patients. Some patients have benefited also from plasma exchange (PLEX) (26, 27). The effects of IVIg and immunoadsorption have been usually unsatisfactory. However, in many of these studies, there has been a long delay from symptom onset to treatment (11, 26). We used immunoadsorption successfully in patient 3. CSF-filtration has also been tried, however with a long delay from symptom onset (11). Second line therapy usually includes cyclophosphamide and rituximab (11, 26). We administered rituximab as second line therapy but not cyclophosphamide in view of its adverse effects in young female patients. Other immunosuppressive agents such as azathioprine and mycophenolate mofetil (MMF) have often been tried (11) with varying results, as also in our patients. Moreover, natalizumab has been tried to block T-cell entry into the CNS (11). In one case report, GAD-epilepsy was successfully managed with basiliximab (28); this was attributed to a reduction in the numbers of activated T-cells via interleukin-2 receptor blockade. Rituximab has an indirect inhibiting effect on pathogenic T-cells (29) which could in part explain its effect as the pathology of GAD-epilepsy seems to be mediated by cytotoxic T cells (4).

It is generally accepted that immunotherapy in GAD-epilepsy should be initiated as soon as possible (15), however there is no clear evidence defining when immunotherapy will no longer be effective. In many previous studies, there has been a long delay to diagnosis and immunotherapy initiation. For example, when there was a 4.5 (±0.4) year delay in immunotherapy, only every fifth patient showed any improvement (26). Furthermore, when the median disease duration was 18 months, it was reported that treatment results were poor (11).

Our results suggest that one obtains optimal results when immunotherapy is initiated during the early stages of acute immunoactivation when no brain MRI changes are yet visible as was seen with patients 1 and 3. In some case reports it has been shown similarly that early initiation of immunotherapy provides complete seizure freedom (30). Thus, there is convincing evidence that early immunotherapy can be effective in the first stage of GAD-epilepsy.

In the second stage of GAD-epilepsy, there is already irreversible brain tissue damage causing refractory epilepsy as was observed in patient 2 and in many previous case series which have demonstrated a poor treatment response to immunotherapy (11). However, we could show that the already developed brain MRI abnormalities could be resolved after regular immunotherapy. In some case reports, immunotherapy has also achieved a similar resolution of the MRI abnormalities (16, 31). There is one case report describing the empirical initiation of MP, IVIg, plasmapheresis, rituximab and cyclophosphamide in refractory status epilepticus which later proved to be GAD-epilepsy (16). After 1 month, that patient was almost symptom-free with only occasional breakthrough seizures with regular rituximab infusions and 5 AEDs with resolution of the MRI abnormalities (16). This evidence is suggesting that even during the second stage of GAD-epilepsy, immunotherapy can reverse brain tissue damage and possibly prevent a more severe clinical course of GAD-epilepsy. However, in this stage, the management of GAD-epilepsy shifts from immunotherapy to managing the refractory epilepsy.

In third stage of GAD-epilepsy, there already has occurred permanent progressive damage, possibly hippocampal sclerosis and permanent cognitive symptoms. One of our patients with late GAD-epilepsy diagnosis had developed hippocampal sclerosis, as has often been shown before (6) as the cytotoxic process seems to initially involve limbic areas (4). Moreover, widespread white matter changes have been detected in GAD-LE (7) suggesting that there is also a more widespread pathology. Late immunotherapy in refractory GAD-epilepsy had little effect, which is in line with previous evidence (26). However, there is one case report which claimed that PLEX exerted a clear effect 7 years after symptom onset even though MP and IVIg had no effect (27) and in one study, basiliximab showed temporal resolution of seizures also 7 years after diagnosis (28). For these reasons, immunotherapy should be tried at least shortly, even in late GAD-epilepsy diagnosis.

AED selection in GAD-epilepsy is undertaken according to the normal clinically accepted principles in attempts to achieve maximum seizure control and tolerability (15). Only a few GAD-epilepsy patients become seizure-free exclusively with AEDs (32). AEDs also have immunomodulatory effects which could in part explain their effect on the autoimmune epilepsies (32). All but one of our patients required multiple AEDs. However, after the symptoms were controlled with immunotherapy, some AEDs could be discontinued. Moreover, we recommend that when immunotherapy is no longer effective, it is advisable to concentrate on the management of epilepsy. One of our patients responded well to VNS which has not been shown previously in GAD-epilepsy patients. Epilepsy surgery was performed on two of our patients but it exerted no clear effect on seizure levels and this resembles the situation in other GAD-epilepsy patients (6). The better response to VNS than to epilepsy surgery might be because of the diffuse pathology in GAD-epilepsy (7). In all three of our refractory patients, however, the epileptic focus was eventually bilateral, pointing to an insidious continuing cytotoxic process. It seems that early immunotherapy can halt the destructive process and epilepsy surgery could be avoided.

A clear limitation of our study is the low number of patients and the retrospective nature of the study design. However, GAD-epilepsy is a rare entity and large patient materials are difficult to obtain. Moreover, our patients showed varying symptoms. Previously only GAD-TLE or GAD-LE patients have been studied. In this study, we combined GAD-epilepsy patients with different presentations and also the diagnoses had been made with varying delays. However, this also shows that GAD-epilepsy should be suspected in many different clinical scenarios and we have provided new evidence on the timing of the treatments.

In conclusion, these results highlight the importance of early detection of GAD65 antibodies in refractory epilepsy as immunotherapy can be effective during the early stages of the disease and it can possibly prevent the development of permanent brain tissue damage.

## Ethics statement

A case report is a medical/educational activity that does not meet the DHHS definition of research, which is: a systematic investigation, including research development, testing and evaluation, designed to develop or contribute to generalizable knowledge. Therefore, the activity does not have to be reviewed by a IRB.

## Author contributions

K-MM, AH, AB, JP conceived and designed the study. K-MM, AH, AB, JP analyzed the data. AB analyzed MRI images. K-MM, AH, AB, JP wrote the paper.

### Conflict of interest statement

The authors declare that the research was conducted in the absence of any commercial or financial relationships that could be construed as a potential conflict of interest.
